# The Impact of COVID-19 pandemic on pediatric surgery practice: A cross-sectional study

**DOI:** 10.1016/j.amsu.2020.09.020

**Published:** 2020-09-15

**Authors:** . Gunadi, Yofizal Idham, Vincentia Meta Widya Paramita, Aditya Rifqi Fauzi, Andi Dwihantoro, Akhmad Makhmudi

**Affiliations:** Pediatric Surgery Division, Department of Surgery, Faculty of Medicine, Public Health and Nursing, Universitas Gadjah Mada/Dr. Sardjito Hospital, Yogyakarta 55281, Indonesia

**Keywords:** COVID-19 pandemic, Elective and emergency surgeries, Indonesia, Outbreak, Outpatient services, Pediatric surgery practices

## Abstract

**Background:**

Since the COVID-19 pandemic was declared by the World Health Organization on March 11, 2020, routine clinical practices were affected, including pediatric surgery services. We aimed to compare pediatric surgery practices, including the number and types of surgery, either elective or emergency surgeries and outpatient services, before the outbreak and during the COVID-19 pandemic in our institution.

**Material and methods:**

We retrospectively compared pediatric surgery practices, including elective and emergency surgeries, and outpatient services between the previous one-year period (March 2019–February 2020), the last three months of that period (December 2019–February 2020) before the outbreak, and the three months (March–May 2020) during the COVID-19 pandemic in our hospital.

**Results:**

The frequency of elective surgeries during the pandemic was lower than during the last three months before the outbreak: 61 vs. 18 (~3-fold), 19 vs. 13 (~1.5-fold), 19 vs. 5 (~4-fold), and 30 vs. 15 (~2-fold) for digestive, neonate, urology and oncology cases, respectively. No laparoscopic procedures were performed during the pandemic compared with the one-year period before the outbreak (0 *vs.* 16 cases). The frequency of all emergency pediatric procedures before and during the COVID-19 pandemic was similar (29 *vs.* 20 cases, respectively). Moreover, a declining trend was also clearly apparent in the outpatient services during the pandemic compared with before the outbreak, both in the new and the established patients.

**Conclusions:**

The pediatric surgery practices in our institution have been severely affected by the COVID-19 pandemic, including elective and outpatient services. This setback needs a comprehensive strategy to avoid morbidity from the neglected elective surgeries during the pandemic, including the proper comparison between the real risk of COVID-19 cross-infection and the benefits of elective procedures.

## Highlights

• Since the COVID-19 pandemic was declared by the World Health Organization on March 11, 2020, routine clinical practices were affected, including pediatric surgery services.
• The pediatric surgery practices have been severely affected by the COVID-19 pandemic, including the elective and outpatients' services.
• This setback needs a comprehensive strategy to avoid morbidity from the neglected elective surgeries during the pandemic, including the proper comparison between the real risk of COVID-19 cross-infection and the benefits of elective procedures.

## 1 Introduction

The World Health Organization (WHO) declared COVID-19 as a worldwide pandemic on March 11, 2020 [1]. The first two cases of COVID-19 were identified in Indonesia on March 2, 2020 [2], while the first case in the Special Region of Yogyakarta Province was announced on March 15, 2020 [3]. The total population of Yogyakarta Province in the beginning of June 2020 is 3,882,288, while the pediatric population is 997,159 [4]. Eventually, the Special Region of Yogyakarta was indicated to have local transmission of COVID-19 on April 22, 2020. Until September 6, 2020, the total number of confirmed patients with COVID-19 in Yogyakarta was 1557 cases and 46 deaths [3]. Moreover, instead of the lockdown, our provincial government applied the emergency response for the COVID-19 policy [3].

Our hospital is a tertiary referral hospital that primarily serves urban and rural populations from the Special Region of Yogyakarta Province, Indonesia [[5], [6], [7]]. Accordingly, our hospital was assigned by the Ministry of Health of the Republic of Indonesia as a referral hospital for the management of patients with COVID-19 in Yogyakarta Province during the pandemic by a public emergency edict. Recently, several studies showed that the COVID-19 pandemic affected pediatric surgery services [[8], [9], [10], [11]]; however, the reports described the effect of the pandemic on pediatric surgery practice in general [8,9] but did not specifically analyze the number and type of surgeries affected by the outbreak and only provided editorial comments [10] or perspectives [11]. Therefore, we aimed to compare pediatric surgery practices, including the number and types of surgery, either elective or emergency surgeries and outpatient services, before the outbreak and during the COVID-19 pandemic in our institution.

## 2 Material and Methods

### 2.1 Patient samples

We retrospectively compared the pediatric surgery practices elective and emergency surgeries and outpatient services between the previous one-year period (March 2019–February 2020), the last three months of that period (December 2019–February 2020) before the outbreak and the three months (March–May 2020) during the COVID-19 pandemic in our hospital. We chose the last three months before the outbreak (December 2019 – February 2020) to compare the pediatric surgical burden between the first three months of the pandemic and just before the outbreak in our institution. Moreover, in Indonesia, there are no seasonal variations, and the all school schedule from elementary until undergraduate school is started between July and August every year.

We classified pediatric surgery patients' services in our hospital into four categories: digestive, neonates, urology and oncology. Moreover, we also defined those patients’ services into two additional categories: laparoscopic *vs.* nonlaparoscopic surgery.

The Medical and Health Research Committee of our institution approved this study (KE/FK/0653/EC/2020). Written informed consent was obtained from all parents of the pediatric patients who visited/admitted to our hospital during the previous one-year period (March 2019 – February 2020) before the outbreak and the three months (March – May 2020) during the COVID-19 pandemic. The work has been reported in line with the STROCSS criteria [12].

### 2.2 COVID-19 assessment

The diagnosis of COVID-19 using real-time polymerase chain reaction (RT-PCR) in the Special Region of Yogyakarta Province was conducted in five laboratories, including our institution.

## 3 Results

### 3.1 Elective surgeries

**Fig. 1:**
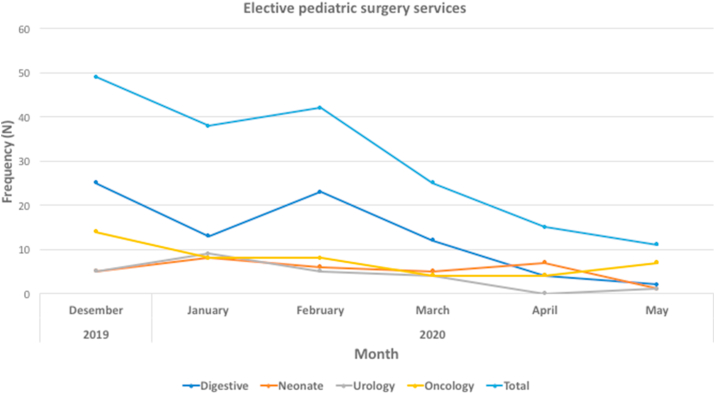
Comparison of elective pediatric surgeries performed in our institution before and during the COVID-19 pandemic from December 2019 to May 2020. The frequency of all elective surgeries during the pandemic was approximately 1.5 – 4-fold lower than those of the last three months before the outbreak, including digestive, neonate, urology and oncology cases.

First, we compared the frequency and types of elective surgeries performed in our hospital. There was a significant decline in the number of elective surgeries in each type of surgery (Table 1 and Fig. 1). The frequency of elective surgeries during the pandemic was lower than those of the last three months before the outbreak: 61 *vs.* 18 (~3-fold), 19 *vs.* 13 (~1.5-fold), 19 *vs.* 5 (~4-fold), and 30 vs. 15 (~2-fold) for digestive, neonate, urology and oncology cases, respectively (Table 1).

**Table 1:** Comparison of elective pediatric surgeries performed in our institution before and during the COVID-19 pandemic.

	Digestive (n, %)	Neonate (n, %)	Urology (n, %)	Oncology (n, %)	Total (n, %)
Mar 2019 – Feb 2020	210 (46.4)	79 (17.4)	58 (12.8)	106 (23.4)	453 (100)
Dec 2019 – Feb 2020	61 (47.3)	19 (14.7)	19 (14.7)	30 (23.3)	129 (100)
Mar – May 2020	18 (35.3)	13 (25.5)	5 (9.8)	15 (29.4)	51 (100)

The numbers in parentheses indicate percentages.

Subsequently, we compared the frequency of each surgical procedure of elective services according to the disease (Table 2). Almost all surgical procedures during the pandemic were fewer than the last three months before the outbreak, such as pull-through for Hirschsprung disease (3 *vs.* 15 cases), anoplasty and stoma closure for anorectal malformation (1 *vs.* 9), chordectomy and urethroplasty (1 *vs.* 5) and excision and bleomycin sclerotherapy for lymphangioma (5 *vs.* 12) (Table 2).

**Table 2:** Comparison of the frequency of each procedure of elective services according to the disease before and during the COVID-19 pandemic.

	Mar 2019 – Feb 2020 (n)	Dec 2019 – Feb 2020 (n)	Mar – May 2020 (n)
Hirschsprung diseaserowhead Full-thickness rectal biopsyrowhead Stomarowhead Soave pull-throughrowhead Duhamel pull-throughrowhead Transanal endorectal pull-throughrowhead Transanal Swenson-like pull-throughrowhead Kimura colonic patchrowhead	53 19 1 16 19 8 1	7 3 0 7 5 3 0	7 1 0 0 3 0 0
Anorectal malformationrowhead Colostomyrowhead Anoplastyrowhead Stoma closurerowhead	11 28 13	1 6 3	0 1 0
Hepaticojejunostomy Roux-en-Y for choledochal cystrowhead	2	0	0
Kasai procedure for biliary atresiarowhead	6	4	1
Kimura procedure for duodenal atresiarowhead	10	5	4
Esophageal atresiarowhead Gastrostomyrowhead Primary anastomosisrowhead	6 3	1 0	1 0
High ligation for inguinal herniarowhead	26	8	6
High ligation for hydrocelerowhead	5	1	0
Hypospadiasrowhead Chordectomyrowhead Urethroplastyrowhead	7 11	3 2	1 0
Circumcisionrowhead	18	8	1
Lymphangiomarowhead Excisionrowhead Bleomycin sclerotherapyrowhead	10 27	4 8	1 4
Excisional biopsy for rhabdomyosarcomarowhead	1	0	0
Excisional biopsy for neuroblastomarowhead	4	1	0
Nephrectomy for Wilms' tumor	5	0	1

Moreover, none of the laparoscopic procedures were performed during the pandemic compared to the previous one year before the outbreak (0 *vs.* 16 cases: laparoscopic anoplasty = 3, laparoscopic appendectomy = 3, laparoscopic orchiopexy = 4, laparoscopic unroofing of splenic cyst = 1, laparoscopic-assisted transanal endorectal pull-through = 2, laparoscopic high ligation = 1, and laparoscopic cholecystectomy = 2).

### 3.2 Emergency procedures

**Fig. 2:**
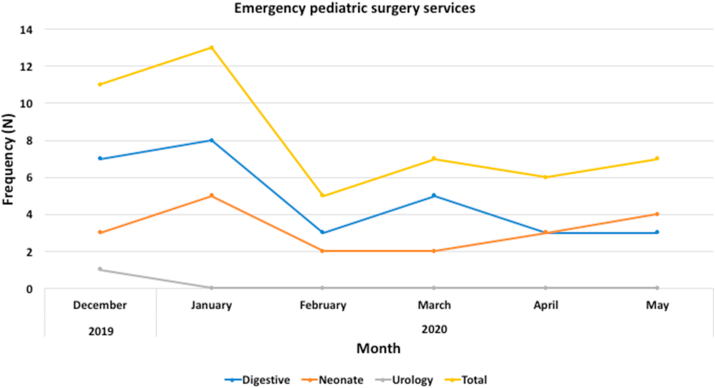
Comparison of emergency pediatric surgeries conducted in our hospital before and during the COVID-19 pandemic from December 2019 to May 2020.

The frequency of emergency pediatric surgical procedures before and during the COVID-19 pandemic was similar (29 *vs.* 20 cases, respectively) (Table 3 and Fig. 2).

**Table 3:** Comparison of emergency pediatric surgeries performed in our hospital before and during the COVID-19 pandemic.

	Digestive (n, %)	Neonate (n, %)	Urology (n, %)	Total (n, %)
**Mar 2019 – Feb 2020**	54 (52.4)	44 (42.7)	5 (4.9)	103 (100)
**Dec 2019 – Feb 2020**	18 (62.1)	10 (34.5)	1 (3.4)	29 (100)
**Mar – May 2020**	11 (55)	9 (45)	0	20 (100)

The numbers in parentheses indicate percentages.

Next, we compared the frequency of each surgical procedure of emergency services according to the disease (Table 4). Almost all emergency procedures before and during the outbreak were similar, including laparotomy appendectomy (6 *vs.* 4 cases), laparotomy milking procedure for intussusception (3 *vs.* 2 cases), and colostomy for anorectal malformation (2 *vs.* 4 cases).

**Table 4:** Comparison of the frequency of each surgical procedure of emergency services according to the disease before and during the COVID-19 pandemic.

	Mar 2019 – Feb 2020 (n)	Dec 2019 – Feb 2020 (n)	Mar – May 2020 (n)
Laparotomy for perforated appendicitis	24	6	4
Laparotomy milking procedure for intussusception	8	3	2
Abdominal wall defect repair	7	2	1
Anorectal malformationrowhead Colostomyrowhead Anoplastyrowhead	13 5	2 0	4 1
Laparotomy repair of congenital diaphragmatic hernia	7	1	0
Pyloromyotomy for hypertrophic pyloric stenosis	5	0	1
Resection and primary anastomosis for jejunoileal atresia	1	0	1
Ladd's procedure for malrotation	1	1	0
Orchiopexy for testicular torsion	5	1	0

### 3.3 Outpatient services

**Fig. 3:**
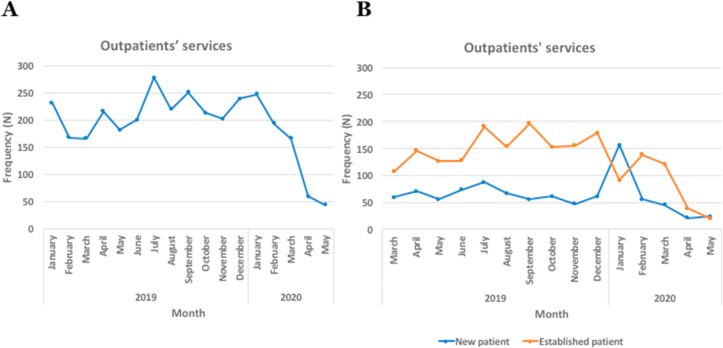
Comparison of outpatient services in our hospital before and during the COVID-19 pandemic from March 2019 to May 2020. The frequency of all outpatients' (A) and new and established outpatients' (B) services during the pandemic was lower than those of the previous year before the outbreak.

A declining trend was clearly apparent in the outpatients’ services during the pandemic compared with before the COVID-19 outbreak (Fig. 3A). Next, we divided the outpatient services into new and established patients. Similar decreasing trends were obtained (Fig. 3B).

## 4 Discussion

We are able to show the effect of the COVID-19 pandemic on decreasing the number of pediatric surgical services in our institution, including elective and outpatient services. These declining trends might be related to the fact that all non-urgent elective surgeries were temporarily suspended to ensure adequate hospital capacity to respond to the rapid spikes in COVID-19 cases and decrease the risk of nosocomial transmission of COVID-19 infection. This strictly enforced policy was applied in almost all hospitals around the world that were affected by the COVID-19 pandemic, including Australia [13], Finland and other Nordic countries and the United States of America (USA) [9]. Moreover, the relative lack of medical resources due to the increasing number of patients with COVID-19 and the accompanying economic downturn might also influence the management decisions for pediatric surgery patients [8]. It should be noted that delay of surgery for “time-sensitive” and urgent diseases in children might affect their growth, development, and quality of life [8]. Therefore, we still performed elective surgeries for neonates and oncology cases. However, our findings showed a declining trend in neonates and oncology cases as well, with approximately 1.5 – 2-fold fewer of these surgical procedures performed during the outbreak compared to before the pandemic (Table 2). This difference might be related to the fact that many families were worried about whether it is safe to bring their children to the hospital [9,14].

Although still considered controversial, we avoided laparoscopic procedures during the pandemic to minimize the risk of aerosol transmission, as recommended by a previous report [15]. Several methods have been proposed to reduce the risk of cross-infection of COVID-19 during laparoscopic surgery: a) properly decreasing the pressure of pneumoperitoneum; 2) avoiding the leakage of gas from the trocar places; and 3) gradually eliminating the aerosol via aspirator after pneumoperitoneum [8].

While our data showed declining trends in the elective surgical cases, the number of emergency procedures did not appear to be significantly affected by the pandemic (Table 3). Our findings were similar to those of a previous report [9]. The number of laparotomies performed for perforated appendicitis was similar before and during the pandemic (Table 3). In the USA, some hospitals applied non-operative management for acute appendicitis, while other institutions continued to perform routine appendectomies [9]. Notably, no consensus has been established yet for the management of acute appendicitis during the COVID-19 pandemic. The choice between conservative treatment and emergency surgical procedures with appendectomy depends on the resources of each institution [9].

Our government has applied restrictions on travel between provinces and/or cities in the earlier period of the pandemic. Moreover, many families were worried about whether it is safe to bring their children to the hospital, as noted by other reports [9,14]. These facts might affect our results.

Since the COVID-19 pandemic might end in months, on June 1, 2020, our government announced a “new normal” policy to start implementing adaptations of the public's daily activities to the COVID-19 pandemic, including updated changes in health care services [16]. Accordingly, our pediatric surgery division in our hospital has tried to normalize our services as follows: each week, one major surgery will be performed every Tuesday, while two or three minor procedures will be scheduled and conducted on another working day. These policies started June 8, 2020.

Furthermore, it has been recommended that all pediatric surgeons should contact each other and benefit from the positive, synergistic effects from sharing experiences and best practices during the pandemic with other colleagues [9].

Although several studies showed that the COVID-19 pandemic affected pediatric surgery services [[8], [9], [10], [11]], our study has the following strengths: we specifically analyzed the number and type of surgeries affected by the outbreak (*vs.* described the effect of the pandemic on pediatric surgery practice in general [8,9] *vs.* editorial comments [10] *vs.* perspectives [11]). Notably, our findings are limited to one pediatric surgical center. These facts should be considered during the interpretation of our study.

Our findings suggest that a comprehensive strategy is needed either by the hospital or health district or regional pediatric surgeon association to avoid morbidity from neglected elective surgeries during the pandemic, including the proper comparison between the real risk of COVID-19 cross-infection and the benefits of elective procedures.

## 5 Conclusions

The pediatric surgery practices in our institution have been severely affected by the COVID-19 pandemic, including elective and outpatient services.

## Conflicts of interest

No potential conflict of interest relevant to this article was reported.

## Funding

This study was funded by Indonesian Ministry of Research and Technology/National Agency for Research and Innovation.

## Consent

Written informed consent was obtained from the parents before joining the study. A copy of the written consent is available for review by the Editor-in-Chief of this journal on request.

## Provenance and peer review

Not commissioned, externally peer reviewed.
